# Advanced Hydrogels in Breast Cancer Therapy

**DOI:** 10.3390/gels10070479

**Published:** 2024-07-19

**Authors:** Xiangyu Gao, Benjamin R. Caruso, Weimin Li

**Affiliations:** 1 Department of Translational Medicine and Physiology, Elson S. Floyd College of Medicine, Washington State University, Spokane, WA 99202, USA; 2 Doctor of Medicine Program, Elson S. Floyd College of Medicine, Washington State University, Spokane, WA 99202, USA; benjamin.caruso@wsu.edu

**Keywords:** hydrogel, breast cancer, tissue engineering, regenerative medicine, 3D bioprinting, 3D culture, organoids, drug delivery, chemotherapy, immunotherapy

## Abstract

Breast cancer is the most common malignancy among women and is the second leading cause of cancer-related death for women. Depending on the tumor grade and stage, breast cancer is primarily treated with surgery and antineoplastic therapy. Direct or indirect side effects, emotional trauma, and unpredictable outcomes accompany these traditional therapies, calling for therapies that could improve the overall treatment and recovery experiences of patients. Hydrogels, biomimetic materials with 3D network structures, have shown great promise for augmenting breast cancer therapy. Hydrogel implants can be made with adipogenic and angiogenic properties for tissue integration. 3D organoids of malignant breast tumors grown in hydrogels retain the physical and genetic characteristics of the native tumors, allowing for post-surgery recapitulation of the diseased tissues for precision medicine assessment of the responsiveness of patient-specific cancers to antineoplastic treatment. Hydrogels can also be used as carrier matrices for delivering chemotherapeutics and immunotherapeutics or as post-surgery prosthetic scaffolds. The hydrogel delivery systems could achieve localized and controlled medication release targeting the tumor site, enhancing efficacy and minimizing the adverse effects of therapeutic agents delivered by traditional procedures. This review aims to summarize the most recent advancements in hydrogel utilization for breast cancer post-surgery tissue reconstruction, tumor modeling, and therapy and discuss their limitations in clinical translation.

## 1. Introduction

Breast cancer is defined as a malignant neoplasm in the breast. Of all cancers, it has the highest incidence in women globally. In the United States, about 310,720 new cases of invasive breast cancer will be diagnosed in women in 2024 [1]. As the second leading cause of cancer death after lung cancer in women, breast cancer claims the lives of about 1 in 40 [1].

The standard treatment for breast cancer involves a combination of surgical, drug, and radiation interventions, depending on tumor grade, stage, and the patient’s physical condition. Surgical interventions span from breast-conserving lumpectomies to radical mastectomy. Radiation therapy (or radiotherapy) applies to unresectable or metastatic breast cancer. Pharmacological intervention, depending on drug categories and molecular characteristics, can be chemotherapy, immunotherapy, hormone therapy, or targeted therapy.

Despite being a golden standard for treatment, surgical interventions, particularly mastectomies, are associated with elevated rates of depression, anxiety, stress, and body image disturbances for breast cancer survivors [2,3]. Pharmacological drug interventions may be less invasive than surgery but face the challenges of multidrug resistance, which accounts for 90% of cancer deaths [4]. The resistance mechanisms include drug efflux, genetic adaptations, enhanced epigenetic alterations, tumor heterogeneity, and tumor microenvironment (TME) factors [4]. Additionally, exposure to chemotherapeutics, such as taxanes, platinum analogs, and antitumor antibiotics, causes a variety of acute and chronic adverse effects [5]. These limitations underscore the great need for alleviating the adverse effects of these therapies and improving their therapeutic benefits.

**Figure 1:**
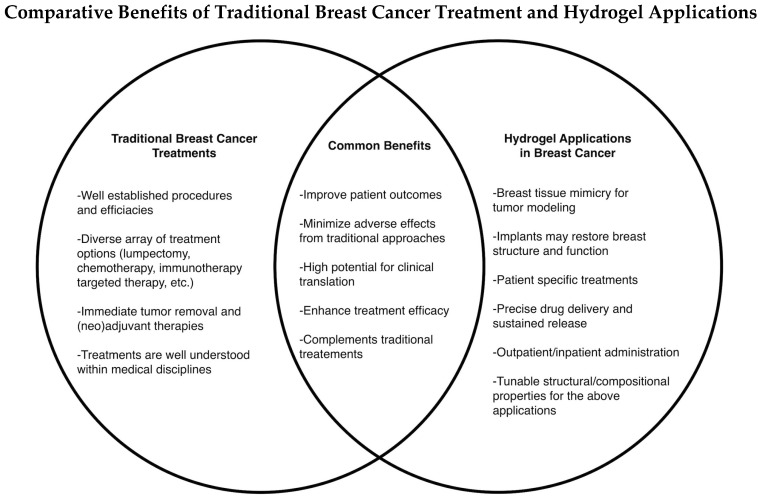
Hydrogel technology complements traditional breast cancer treatment. With the advent of the “engineering era”, applying engineered hydrogels in breast cancer modeling, post-surgical reconstruction, and drug delivery will greatly enhance the capabilities of breast cancer treatment and improve the quality of cancer patients’ lives.

A technology with increasing potential to address the shortcomings of current clinical therapies is hydrogels, a class of polymeric materials with high water content, elasticity, and 3D porous network structures. Their physical resemblance to living tissues has raised significant attention in biomedical applications, particularly in medicine [6,7]. They are candidate breast implants post-mastectomy to restore breast structure and function, potential modeling platforms for patient-specific precision medicine, and drug vehicles to increase the efficacy of antineoplastic treatment and reduce exposure to toxic regimens (Figure 1).

While our previous review presented hydrogel-based scaffolding techniques, modeling of cell–ECM/cell–cell interactions, studying of cellular phenotypes and signaling, and testing of drug sensitivities in 3D cultures [8], the evolving advancements and multidisciplinary nature of hydrogel utilization for breast cancer therapy highlight the need for a specific yet comprehensive review of recent advances in the field. The use of hydrogel in the overall cancer research field has recently been described either in a global or an application-specific way. For example, Li et al. emphasized hydrogel molecular sizes and delivery routes as well as the physical and chemical aspects of hydrogels relative to their potential therapeutic usage [9]. Zhang et al. summarized the applications of hydrogels in immunomodulation studies for cancer treatment and regenerative medicine [10]. Here we specifically summarize the advances of hydrogel applications in breast cancer post-surgical tissue engineering and tumor modeling to facilitate further development of this multidisciplinary technology and its clinical translation.

## 2. Advancements in Hydrogel Implants for Post-Mastectomy Regeneration of Functional Breast Tissues

### 2.1. Common Hydrogels for Breast Tissue Regeneration

#### 2.1.1. Natural Hydrogels

Natural hydrogels are biocompatible and can be derived from animals, plants, algae, or donor human tissues [7,8,11]. Collagen, the most abundant extracellular matrix (ECM) protein in the human breast with acini formation-supporting properties [12], is a master hydrogel for bioengineering and regenerative medicine studies of the human breast. Collagen and its derived gelatin not only mimic the physical properties of the breast ECM but also promote adipocyte proliferation [13] and human breast epithelial cell ductal branching and lobular expansion [14].

Methacrylamide-modified gelatin (GelMA) has been widely used for diverse tissue engineering applications due to its biocompatibility, biodegradability, and modality. The methacrylamide functional groups allow the GelMA molecule to undergo cross-linking for the creation of a methacryloyl backbone, which gives GelMA stability at physiological temperature and allows fine-tuning of mechanical properties [15,16].

Using 3D-printed technology, GelMA can be combined with CaSiO_3_ (CS), a bioactive ion-releasing medium that has been shown to promote adipogenesis and angiogenesis, to form composite scaffolds with customizable architecture for personalized breast reconstruction [17]. This composite scaffold supported adipogenesis and effectively stimulated the vascularization of adipose tissues in vitro and in vivo under the skin of mice. In another study, GelMA loaded with adipose tissue-derived stem cells (ASCs) were combined with methacrylated κappa-carrageenan (CarMA) via 3D printing [18,19]. CarMA is a natural-origin polymer that closely mimics the glycosaminoglycan structure, one of the important constituents of mammalian native tissues ECM [20]. The combined platform exhibited structural stability and mechanical properties akin to native breast tissues and cell viability and proliferation rates similar to those in GelMA scaffolds. ASCs were able to differentiate into adipogenic lineage on the hydrogel blend scaffolds, although their differentiation potential was lower in GelMA combined with CarMA [21]. With a slight variation from GelMA, Zhu et al. generated an injectable nontoxic foamed GelMA (f-GelMA) hydrogel using gas-foaming and light-cured techniques [21]. The f-GelMA acted as a cell carrier and promoted adipose-derived stem cell spheroid (s-ADSC) adipogenic differentiation, anti-apoptosis ability, and tissue graft vascularization in mice.

Despite its potential for versatile formulations, GelMA generation results in high levels of free radicals that damage the cells in the culture and create more heterogeneous polymer networks. To circumvent these shortcomings, a photo-click scaffold was produced using norbornene-functionalized gelatin (GelNB) and thiolated gelatin (Gel-SH) [22]. This method generates porous scaffolds with more homogenous networks, augmented cell attachment and proliferation, and fewer free radicals compared to the GelMA model. Gelatin hydrogels can also be integrated with 3D-printed encapsulated filaments with alginate-based microbeads [23]. This forms microstructures that resemble the microarchitecture of human fatty tissues and microvessels, allowing for vascularization through anastomosis with the patient’s own blood vessels which is crucial for breast adipose reconstruction [24]. The scaffold’s porous structure with hollow channels facilitates nutrient and oxygen diffusion, promoting cell viability and tissue integration. Natural hydrogels have the advantages of good biocompatibility with native tissues, having native tissue mechanical, structural, and mechanical properties, and supporting tissue cell survival and growth for tissue regeneration purposes. However, their scalability, long-term stability, mechanical performance, production expenses, and in vivo induction of immune responses need to be further evaluated. Integration of synthetic hydrogels into natural gels can be a solution to resolve some of the disadvantages of natural hydrogels in translational applications.

#### 2.1.2. Synthetic Hydrogels

Synthetic hydrogels are usually made from non-natural polymers, such as poly(ethyhilene glycol) (PEG), poly(lactic-co-glycolic acid) (PLGA), polycaprolactone (PCL), polyacrylamide (PAM), polyvinyl alcohol (PVA), and Pluronic F127 (PF) [6,25,26,27,28]. They have been explored for breast implantation due to their tunable properties, ease of synthesis [6,28] and higher mechanical stability and durability compared to natural polymers [27]. Their controllable chemical and physical flexibility allow for the achievement of optimal porosity, surface characteristics, and degradation rate, with low variability between models [29]. However, biocompatibility challenges prompt the necessity to enhance their suitability for long-term use in breast tissue engineering and regeneration [7,29,30].

Several recent advancements have been made with PCL-based hydrogels. They can be fabricated to mimic tissue composition and characteristics in several ways. Mohseni et al. printed patient-specific 3D porous scaffolds using medical grade PCL (mPCL) hydrogel and fused filament fabrication (FFF) [31]. The design addresses biomechanical and biological requirements for large-volume tissue regeneration. The external structure provides mechanical stability while the internal structure provides porosity and interconnectivity for tissue formation. Meng et al. developed a technique that allows for tuning of the elastic properties and flexibility of PCL scaffolds using selective laser sintering and sinusoidal filament networks [32], with a goal of fabricating biodegradable scaffolds with mechanical properties comparable to various native soft tissues. Using a similar approach, additively 3D-printed mPCL scaffolds were implanted under the panniculus carnosus muscle along the flanks of the pig for scaffold-guided breast tissue engineering (SGBTE) [33]. Clinically relevant volumes of soft tissues were obtained without wound complications in a period of 12 months, implying a translational potential of the technology.

PCL hydrogels can be combined with other biomaterials for better tissue integration. Griffin et al. made scaffolds with varying pore architectures using PCL and Fused Deposition Modeling (FDM) [34]. They coated the scaffolds with platelet-rich plasma (PRP) to enhance 3T3-L1 adipocyte proliferation. The scaffold with compressive properties like human breast tissues had 40% porosity. The PRP coating enhanced adipocyte formation, tissue integration, and vessel formation in mice. Jwa et al. fabricated PCL scaffolds in the presence or absence of collagen or breast tissue fragments and implanted the scaffolds in breast-defective rat [35]. After 6 months of implantation, the restoration of breast tissues and the expression of collagen were observed in the PCL scaffolds. The addition of collagen to the PCL scaffolds increased fibrous tissues and decreased inflammation. However, neither collagen nor breast tissue fragment addition to the PCL scaffolds restored breast soft tissues in the study. Despite this, the approach showed the potential of repairing tissue defects after partial mastectomy.

Ouyang H et al. created hydrogels with poly(hydroxyethyl methacrylate) (PHEMA) to mimic breast tissues [36]. By mixing hydroxyethyl methacrylate (HEMA) with maleic acid (MA), they reinforced hydrogen bonds and stopped phase separation. These hydrogels have great mechanical properties, with a tensile strength of up to 420 kPa, a fracture strain of 293.4%, a tensile modulus of 770 kPa, and a toughness of 0.86 MJ/m^3^. Their compression modulus is similar to that of silicone breast prostheses, making them feel natural. They also have excellent self-recovery and fatigue resistance, keeping their strength after 1000 compression cycles. Lab and animal tests show they are biocompatible and stable, making them a promising option for breast reconstruction implants [36].

Recently, a synthetic hydrogel composed of polyvinyl alcohol, collagen, and PLGA copolymer/polycaprolactone/gelatin (PVA/COL/PPG) was created to study breast reconstruction [37]. The incorporated PPG nanofibers formed amide bonds with PVA and COL in the composite scaffold that were enhanced by 1-ethyl-(3-dimethyl aminopropyl) carbodiimide (EDC) and N-hydroxysuccinimide (NHS) cross-linking, leading to improved mechanical strength, structural integrity, thermal stability, and cell adhesion, proliferation, and 3D growth. This platform overcomes PVA hydrogel’s limitations of missing bioactive factors and poor structural stability. Depending on the polymers or chemical materials used, synthetic hydrogel production can be complex and cumbersome. While synthetic gels are disadvantageous in biocompatibility, they may not trigger immune system overresponse to reject the synthetic implants either. In addition, the long-term degradation parameters and translational validations of synthetic hydrogel prostheses in animal models need to be further assessed.

#### 2.1.3. Natural vs. Synthetic Hydrogels

Scaffold provision is a major method by which hydrogels can be applied for breast tissue regeneration. Synthetic hydrogels are particularly versatile in this respect, as they can be made with specific mechanical properties and stability, such as those made from PCL using techniques like fused filament fabrication (FFF) and selective laser sintering [31,32]. Their customizability allows them to be tuned to achieve desired porosity, surface characteristics, and degradation rates for optimal scaffold functions [6,25,26,27,28]. The synthetic nature allows for excellent mechanical properties, durability, and fatigue resistance, which are essential for applications requiring long-term stability and load-bearing capacity, such as breast implants [36]. Natural hydrogels can also fulfill this application by being 3D-printed for creating scaffold structures. Due to their more biomimetic properties, they also can support cell viability and mimic the microarchitecture of human tissues [17,18,19,23,24].

In addition to scaffold provision, the actual integration and tissue regeneration following the implant are other major applications for which natural hydrogels have particular potential. Their ability to closely mimic the ECM of native tissues provides a natural environment for growth and regeneration [12,13]. Their ability to be combined with bioactive materials, such as CaSiO_3_ and methacrylated κappa-carrageenan, allows for the enhancement of adipogenesis and angiogenesis, which are crucial for breast tissue regeneration [17,18,19]. Several techniques, such as using f-GelMA and GelNB, allow for stem cell differentiation and free radical protection [17,18,19,21]. Synthetic hydrogels can also mimic these properties by being combined with other biomaterials such as collagen and PRP to improve tissue integration and functionality, enhancing properties like adipocyte proliferation and tissue formation [34,35].

Various challenges exist in both types of hydrogels. Despite their advantages in biocompatibility, natural hydrogels face challenges such as scalability, long-term stability, production expenses, and potential immune responses that need further evaluation [23,24] (Table 1). Synthetic hydrogels face challenges in biocompatibility and long-term degradation, necessitating further assessment of their suitability for translational applications in breast tissue engineering and regeneration [7,30,31,33,37] (Table 1). Further optimization of both types of hydrogels is necessary for clinical translation in breast tissue regeneration.

### 2.2. Breast Tissue Restoration Techniques

Restoring breast function and aesthetics after lumpectomy or mastectomy involves a range of surgical techniques and approaches tailored to meet the needs of the patients [38]. Currently, limited cosmetic surgical options, such as tissue flaps or fat grafts, exist for post-lumpectomy patients [39]. Following a mastectomy, patients face options that require balancing physical limitations and personal preferences. Immediate breast reconstruction, increasingly popular, offers psychological benefits and improved aesthetic outcomes. However, it entails greater surgical complexity and risks due to combined procedures [40].

Currently, the primary approaches for breast reconstruction are synthetic implants and autologous tissue flaps. Synthetic implants, such as silicone gel-filled or saline-filled bags, are relatively straightforward but may lead to complications like implant contracture and leakage [40]. Autologous reconstruction uses the patient’s own tissues, which involves longer surgeries and recovery, but provides better regeneration [41]. Techniques like the transverse rectus abdominis myocutaneous (TRAM) flap from the abdomen or latissimus dorsi flap from the back are utilized, but they can lead to donor site complications [41]. The deep inferior epigastric artery perforator (DIEP) flap method can have long-term results with a natural appearance and tactile sensation. Alternative flap options such as the profunda artery perforator (PAP) flap and lateral thigh perforator (LTP) flap provide additional choices for patients who may not be suitable candidates for abdominal-based reconstructions due to anatomical constraints or personal preferences [42].

An emerging method for breast reconstruction involves using hydrogels as a platform for autologous fat transplantation, vascularization, and tissue engineering in breast reconstruction. These approaches have not been used in standard clinical care and yet offer a new realm of methods for reconstruction involving the use of hydrogels [28,39,43].

#### 2.2.1. Adipogenesis

The human breast is largely composed of vascularized adipose tissue [41], where adipocytes take up 90% of the tissue volume and less than 15% of cellular content [44,45]. Adipogenic engineering or transplantation is thus essential for breast tissue restoration, which requires scaffolds to support adipocyte survival and proliferation. Natural scaffolds derived from ECM components, such as collagen and hyaluronic acid, or decellularized tissues have been explored for their ability to mimic the natural microenvironment and promote tissue integration in breast reconstruction [46]. These scaffolding matrices serve as soil to grow and reestablish breast tissue cell populations. Various cell types, including preadipocytes, smooth muscle cells, and stem cells that serve as seeds, can be implanted into the natural scaffolds to enhance tissue regeneration and improve aesthetic and functional outcomes [46].

Louis et al. showed a method using collagen microfiber (CMF) bio-ink to encapsulate mature adipocytes, ADSCs, and human umbilical vein endothelial cells (HUVECs) for in vitro adipose tissue regeneration [47]. This approach enables the generation of multilayered constructs with a dense vascularized network resembling in vivo adipose tissue structure and essential for graft survival. Ni et al. demonstrated a bottom-up approach using dual micro-tissues composed of ADSCs and HUVECs on collagen microgels [48]. This approach spawned significantly better adipose tissue regeneration and neo-vessel formation compared to single micro-tissue-based grafts and allows for building a large volume of tissue constructs by assembling micro-tissues [49].

#### 2.2.2. Vascularization

Adipose tissues are highly vascularized. The vasculature in adipose tissues is essential for adipocyte survival in vivo, with special regard to its metabolism, endocrine bioactivities, and for maintaining the tissues’ homeostasis. Vascular-associated cells, such as vascular endothelial cells, smooth muscle cells, and pericytes, account for approximately 70% of adipose tissue cell populations and form fenestrated capillaries around cell clusters [44,48] and are indispensable along with supportive roles from pre-adipocytes, fibroblasts, and hematopoietic cells. Establishing functional vasculatures in adipose breast tissues has been a prominent and challenging topic in the field.

Morrison et al. introduced techniques to address vascularization and tissue regeneration challenges using tissue-engineering chamber models [50]. The approaches create environments that promote blood vessel formation and support adipose tissue growth. Chhaya et al. showed a method involving delayed fat injection into patient-specific scaffolds fabricated using additive biomanufacturing technology [51]. Successful angiogenesis and adipose tissue regeneration were observed in all constructs, with the pre-vascularization plus lipoaspirate group exhibiting outcomes comparable to native breast tissues. Puls et al. introduced a regenerative tissue filler for breast-conserving surgery (BCS) that induces a regenerative healing response characterized by rapid cellularization, vascularization, and progressive breast tissue neogenesis [52]. The collagen-based filler demonstrated translational potential for soft tissue reconstruction, maintaining tissue volume, and inducing complex tissue regeneration without inflammation.

### 2.3. Regulatory Status of Hydrogels for Tissue Regeneration

Hydrogels are rapidly evolving in the clinical setting due to ongoing research of new types and formulations. They are widely implemented in clinical settings for a variety of applications with the most common being used for soft contact lenses [53,54]. It was discussed in a review by Clegg J et al. that over 100 hydrogel products, derived from natural materials, synthetic materials, or their combination, have been approved by the Food and Drug Administration (FDA) and European Medicines Agency (EMA) for medical purposes, particularly tissue regeneration following ocular application [55]. These clinically approved tissue regeneration applications portend to a variety of systems such as the cardiovascular, orthopedic, integumentary, and genitourinary systems are also prominent [55].

For uses specifically in breast implantation, traditional silicone-based implants are the main FDA-approved applications [55]. However, tissue regeneration-focused hydrogels made with chitosan, alginate, and polyethylene glycol, as well as a variety of other natural and synthetic materials have been FDA-approved for other purposes such as cartilage, periodontal, and bone regeneration [54,55]. In particular, a heart failure intervention involving implantation in the ventricular myocardium with a bulk collagen hydrogel made with induced pluripotent stem cell (iPSC) cardiac myocyte is in an active clinical trial with an identifier NCT04396899. This method of regeneration shares similarities with the incorporation of ASCs into GelMA scaffolds for breast tissue regeneration [18,19].

## 3. Breast Cancer 3D Hydrogel Models in Precision Medicine

### 3.1. Breast Cancer 3D-Modeling Using Hydrogels

Modeling breast cancer using 3D hydrogel aims to replicate the tumor microenvironment (TME) by incorporating or directly utilizing bioactive ECM or ECM-mimicking components [56]. For instance, chitosan, alginate, hyaluronic acid, cellulose, collagen, gelatin, and silk fibroin have been used to mimic crucial aspects of in vivo TME to study cell–cell or cell–matrix interactions, gene expression alterations, and tissue structural heterogeneity and complexity [57]. Through formulating the compositions and proportions of bioactive polymers, hydrogel-based systems enable fine-tuning of the mechanical properties and, to some extent, the biochemical properties of native tissue ECM mimicries and cell adhesion dynamics, optimizing cell growth within the 3D TME (Table 2).

Collagens, especially type I collagen, are the most abundant ECM proteins in both normal and cancerous breast tissues [12,58,59,60]. They are essential for the ECM structures and acini structural maintenance [12]. Thus, integration of the major collagen types, such as Type I, II, III, and V collagens, commonly found in human, pig, and mouse breast ECM [12,58,59], in bioprinting or 3D culture platforms may better mimic the TME and induce biologically relevant phenotypes of the cultured cells. Shi et al. demonstrated the use of low-concentration collagen-based bio-inks for bioprinting breast tumor organoids, enabling the creation of vascularized tumor models and advancing drug discovery research [61]. It is worth noting that the elastic moduli of normal or tumoral breast tissues or ECM are important parameters for modeling the native tissues [59,62,63]. While Keller et al. stressed the importance of tissue ECM microstructures in engineering pathophysiologically relevant TME for cell growth and interactions [62], Ruud et al. highlighted the significance of different ECM on cancer cell morphological, proliferation, differentiation, migration, and invasion phenotypes [63]. These native tissue-derived total ECM 3D culture models are fundamentally important as they preserve the compositional and structural properties of native tissues for optimal cellular functions that are important for precision medicine testing. Besides the native ECM- and collagen-based hydrogels that are increasingly applied in breast cancer modeling, there are other types of natural or non-natural hydrogel types that can be used for the same purpose and have been intensively reviewed previously [8,64,65,66,67].

With the advancement of chemical or biochemical engineering, functionalized hydrogels have become popular to be used to address broad or specific biological questions. For instance, Collodet et al. functionalized recombinant spider silk with a cell adhesion motif Arg-Gly-Asp (RGD) from fibronectin to maintain characteristic cell marker expression and support patient-derived cell growth [68]. Similarly, elastin-like recombinamer (ELR) hydrogels, composed of ELR polypeptides with MMP-degradable sequence (HE5) and cell-adhesive motif (RGD), were produced as a breast cancer ECM resemblance to grow cancer cells [69]. High cell viability and proliferation rates as well as notable drug resistance were observed for both tumorigenic and non-tumorigenic breast cells in this system, implicating its potential for drug screening assays. While these functionalized gel systems focus on improving cell viability and mimicking the native ECM microenvironment, the non-human or -mammalian tissue ECM protein aspects of the mimicries are their shortcomings that may affect their translational applications.

Tumors are heterogeneous tissues containing both cancer and stromal cells, such as tumor-associated fibroblasts (TAFs) or macrophages (TAMs). In addition to modifying the ECM-mimicking hydrogels at structural and compositional levels, efforts have been made to improve the cellular diversity in 3D hydrogel culture models so that cancer cell responses to treatment could be better reflected. Pierantoni et al. made enzymatically crosslinked silk fibroin hydrogels to coculture breast cancer cells and fibroblasts [70]. They found that, in addition to ECM modifications exerted by the two types of cells that are different from culturing cancer cells alone, the presence of fibroblasts in the culture seemed to endow chemoresistance to the cancer cells over an extended culture period, a phenotype like that seen in native tumor treatment. Xu et al. made an alginate cryogel model to coculture organoids comprising breast cancer cells and monocyte-induced macrophages, which induced an in vitro immune microenvironment [71]. The direct coculture system supported enhanced organoid growth and cancer-aggressive phenotypes. However, the therapeutic testing potential of this model remains to be demonstrated. Moghimi et al., on the other hand, introduced an integrated 3D bioprinted and microfluidic device to coculture breast cancer cells and normal cells to mimic tumor heterogeneity [72]. This device allows the observation of cell migration in the microfluidic chamber towards the chemoattractant. Again, the use of the platform for drug or precision medicine testing is vague. Future works integrating the different cell populations present in a tumor into advanced 3D hydrogel culture systems may discover cancer cell phenotypes that are closer to those displayed in native tumors and provide upgraded therapeutic testing modalities, where cancer cell resistance to drug treatment could be more pronounced than that exhibited in the single or double cell type culturing models.

Recently, hydrogel models have advanced to ex vivo and patient-derived scaffold (PDS) levels that reflect higher levels of heterogeneity of breast cancer tissues and its complex treatment responses compared to that of single- or double-cell line-based models. Koch et al. showed an ex vivo culture model using star-shaped PEG and maleimide-functionalized heparin hydrogels that support the viability of human mammary tissues for up to 3 weeks, though the epithelial phenotype and hormonal receptors were only maintained for 2 weeks [73]. Gustafsson et al., on the other hand, used decellularized patient tumor tissues as scaffolds to culture breast cancer cells, which exhibited significant resistance to endocrine therapeutics and cell cycle inhibitors compared to that of the cells cultured on 2D surfaces [74]. These advances underscore the importance of incorporating patient-specific features into breast cancer models for personalized and precision therapeutic strategies, which can provide more accurate platforms for studying tumor progression and treatment responses at individual levels.

**Table 2:** Summary of the composition, characteristics, and applications of the different types of hydrogels in breast cancer 3D modeling.

Hydrogel Model	Composition	Characteristics	Applications	Reference
ECM and collagen-based	Type I, II, III, V collagen	Mimics native tissue ECM; preserves compositional and structural properties for optimal cell functions	Bioprinting, 3D culture platforms	[12,58,59,60,61,62,63]
Functionalized	Recombinant spider silk with RGD motif, ELR hydrogels with MMP-degradable sequence and RGD motif	Mimics ECM microenvironment; maintains cell marker expression; enhances patient-derived cell growth and drug resistance	Drug screening assays. Addressing biological questions	[68,69]
Coculture systems	Silk fibroin, alginate cryogels	Incorporates stromal cells such as fibroblasts and macrophages; enhances chemoresistance and cancer cell phenotypes	Studying cell–cell interactions and chemoresistance	[70,71,72]
Ex vivo and PDS	Star-shaped PEG, maleimide-functionalized heparin, decellularized patient tumor tissues	Incorporates patient-specific features; reflects heterogeneity and complex treatment responses	Studying personalized and precision therapeutic strategies	[73,74]

### 3.2. 3D Hydrogel Applications in Antineoplastic Precision Medicine

Despite continuous improvement in antineoplastic regimens, cancer patients often develop resistance to treatments, especially in the case of metastatic disease [75,76,77]. This is largely due to tumor heterogeneity and genomic instability [78,79,80]. 3D culture has high biological relevance in mimicking the TME, maintaining natural cell shapes, heterogeneous interface with medium, and cell–cell junctions that are essential for the cells in the culture to exhibit their natural phenotypes. These properties of 3D culture models are advantageous over 2D cultures for drug testing in vitro. In the last 16 years, 3D organoid models have become a powerful tool for therapeutic screening and precision medicine testing for cancer treatment [81,82]. Addressing the high genomic instability and altered phenotypes of cells grown in 2D cultures, patient-derived organoid (PDO) 3D cultures effectively retain the characteristics of a patient’s original tumor [83,84,85,86,87,88,89]. For instance, breast cancer organoids in hydrogels demonstrated increased chemoresistance compared to those in 2D cultures, like those observed in other 3D culture platforms [58,74,90,91]. This enhanced chemoresistance, resembling that in a native tumor, is mainly rooted in the heterogeneous properties of the 3D tumorous structures at multiple levels, such as native tissue cell genetic, ECM structural, mechanical, or compositional heterogeneity, cellular metabolic, differentiation, proliferation, or molecular activity heterogeneity, and the cell population heterogeneity [8,92]. Another advantage of organoids is their biobankability for extended research or therapeutic testing [93]. The advancements in the versatile biomimetic 3D in vitro tumor models and organoid generation have made the combination of in vivo-like TME and organoids in one system possible and offered unprecedented opportunities for antineoplastic drug evaluations.

Sachs et al. tested breast cancer organoid responses to chemotherapeutic drugs and demonstrated a good match of the treatment responses with that of the respective patients [93]. At the same time, the study highlighted the challenge of testing over the surgically removed tumor-derived organoids as their responses to drug treatment would not be able to map back to the treatment on the patients because of tumor removal. Differential response of organoids derived from different patients to drug treatments was also observed, reflecting a reality of interpersonal differences in response to the same drug treatment and highlighting the importance of personalized medical treatment of human cancers. In a clinical trial study, Divoux et al. proposed to use patient-derived tumor organoid (PDTO) from triple-negative breast cancer (TNBC) patients’ biopsies and test the PDTOs’ responses to chemotherapy and immune checkpoint blocker (ICB) in comparison to the responses of the patients [94]. It will be interesting to see if the TNBC PDTOs would display similar responses to the drug treatment as seen in the studies of Sachs et al. described above. Wu et al. produced PDOs from 75 patients’ biopsies or surgical specimens and cultured in Cultrex basement membrane extract (BME) for treatment testing with chemo, endocrine, targeted, or herb-derived drugs [95]. By comparing with the treatment responses in patients, the authors indeed confirmed the feasibility and the advantage of using PDOs for individualized drug efficacy evaluation and regimen optimization that are very useful for improving therapeutic strategies against recurrent, metastatic, and treatment-resistant breast cancers. In a similar study, Chen et al. established 99 breast cancer samples with drug-resistant and metastatic backgrounds for drug screening [96]. High reproducibility of drug screen using the PDOs was achieved, and multidrug resistance was found in a proportion of the organoid lines, suggesting distinct drug responses across the PDOs from the individual patients. To model microbiome effects on anti-tumor immunity, Shelkey et al. established an immune-enhanced tumor organoid (iTO) model using 4T1 mouse TNBC and spleen-derived immune cells encapsulated in methacrylated collagen and thiolated hyaluronic acid to examine the impact of bacterial metabolites on immune checkpoint blockade response for cancer cell apoptosis induction [97]. Though the system lacks macrophage, which is a major immune respondent, and uses the mouse instead of human breast cancer cells, this study demonstrated a synergistic effect of the immunomodulatory host microbiome analog found in bacteria and ICB on cancer cell apoptosis. Again, the hydrogel-based 3D organoid model was shown to be an effective platform for precision therapeutic testing. To study dynamic interactions between immune cells and PDO, Dekkers et al. developed a BEHAV3D system, which could live-track the efficacy and mode of action of cellular immunotherapy on PDO grown in BME [98]. This model not only showed the potential of using hydrogel-cultured PDOs in maintaining tumor-specific inflammatory features but also highlighted the feasibility of using the system or others alike in modeling cancer cell responses to immunotherapy in a patient-specific way.

Hormone therapies are a major domain of breast cancer treatment in addition to chemotherapy and immunotherapy [99]. Tamoxifen (Nolvadex or Soltamox), fulvestrant, and palbociclib are estrogen receptor blockers used to treat estrogen receptor-positive (ER+) breast cancer, which is a subtype of breast cancer that uses estrogen for cancer cell proliferation and disease progression [100]. These receptor blockers inhibit cancer cell growth and alter the cells’ gene expression. Hogstrom et al. developed a PDO and matching CAF coculture model, where the organoids and cells were derived from hormone receptor-positive breast cancers and cocultured in BME, to study endocrine therapy resistance [101]. They found that their PDO model retained ER expression and ER responsiveness well during prolonged culture periods for over one year. CAF-secreted cytokine-mediated resistance to estrogen receptor antagonist fulvestrant was observed, reminiscent of what happens in cancer patients. In support of hormone therapy testing models, an ER+ breast cancer organoid medium (BTOM-ER), which conserves ER expression, estrogen responsiveness, and dependence as well as sensitivity to endocrine therapy of ER+ PDO, was developed by Oliphant et al. [102]. The medium can promote the generation and maintenance of ER+ breast cancer organoids and, thus, facilitate studies of ER+ cancer cell responses to hormone therapies and improvement of therapeutic regimens. These therapeutic-testing PDO models collectively emphasize the power of bioengineered hydrogel platforms in supporting native tumor-like 3D cancer biology that will transform the future drug screening practice and improve the treatment efficacies of therapeutic regimens.

Though organoid models are promising for precision medicine testing, certain remaining challenges limit their broad clinical applications to benefit patients in the real world. First of all, as realized in the Sachs et al. study discussed above [93], although the surgically removed tumors can be used for drug testing in PDO systems, the model-based treatment regimens are hardly applicable to the patients where the tissues are collected from because there are no tumors in the patients anymore, unless there are recurrences later on, at which point the status of the tumor could be different than the primary tumors. Second, core needle biopsy tumor specimens are alternative options for PDO generation and therapeutic testing. Yet, the small amount and heterogeneity of the tissue samples, which may contain only a small number of cancer, stromal, and immune cells, make it difficult to perform sufficient multidrug testing in PDO cultures and to draw a confident conclusion from the testing. Third, most of the hydrogels currently used for PDO cultures are remote to native tissue ECM that may not provide the TME conditions matching with those in a patient’s tumor. Therefore, further development and optimization of the PDO and other hydrogel-based 3D culture models are necessary for clinical translation of the current hydrogel platforms for precision medicine testing.

## 4. Hydrogels in Antineoplastic Delivery

Hydrogels have drug delivery capabilities and controllable drug release kinetics. These hydrogel properties allow for the accommodation of a wide range of therapeutic agents, including chemotherapeutics and immunosuppressants or immune-promoting agents, facilitating a cascade of therapeutic modalities for integrated cancer treatment approaches [103,104,105]. They are available in various particle sizes, from macrogels (>100 μm) to microgels (0.5–10 μm) and nanogels (<200 nm), and can be administered through multiple delivery routes, such as intravenous injection, in situ implantation, transdermal delivery, oral delivery, pulmonary delivery, and transarterial chemoembolization. These versatilities, tunabilities, and applying flexibilities enable more precise and effective targeting of cancer sites, ensuring continuous and controlled drug delivery while minimizing systemic toxicity and reducing the required drug dosage [104,106,107,108,109,110,111].

The responsiveness to internal and external stimuli of hydrogels also enables the controlled release of anti-cancer agents based on specific TME cues, such as variations in pH, temperature, redox potential, and reactive oxygen levels. This responsive behavior provides room for enhancing therapeutic efficacy while minimizing damage to normal tissues, thereby improving patient outcomes. Injectable hydrogels are also being explored as localized drug delivery methods in cancer therapy, offering targeted drug delivery with reduced forces and increased adaptability to tumor resection cavities. The incorporation of silicate nanoparticles into these hydrogels further expands their biomedical applications, underscoring the versatility and promise of hydrogels in antineoplastic drug delivery [106,107,108,109,110,111].

### 4.1. Hydrogel Delivery of Chemotherapy

Several hydrogels have been explored for chemotherapeutic potential in breast cancer treatment. A common material used is chitosan, a natural cationic and hydrophilic copolymer that can be degraded by human enzymes [112]. This property allows for their mucoadhesive characteristics from interactions with opposite charges, which provides the ability of tissue binding for specific drug delivery [113,114]. In a study by Seo et al., methacrylated glycol chitosan (MGC) hydrogel with extended release of DNA/doxorubicin (DOX) complex was made to serve as a local drug delivery platform [115]. They found patients treated with MGC-DOX exhibited the lowest lung metastasis rate and highest survival rate compared to other groups (no hydrogel and free DNA/DOX complex). Also, Yang et al. created a 3D sponge loaded with cisplatin–chitosan (CS)–calcium alginate microparticles (MPs) for breast regeneration post-resection of cancer [116]. Their results indicated that this composite material could treat hemorrhage through its porous structure, blood absorption capabilities, and enhanced coagulation ascribing to the nature of CS or gelatin. Additionally, as the concentrations of loaded MPs increased, the antitumor efficacy also increased [116]. This highlights the potential of this gel in post-mastectomy breast reconstruction, offering a dual benefit of tumor safety and better regenerative outcomes in postoperative implants. Along the same goals of regeneration and antitumor properties, Shi et al. printed 3D intelligent scaffolds (IS) using PLGA, gelatin, and chitosan loaded with anti-cancer drugs that showed anti-tumor ability for up to 30 days in mice, with effective tumor growth inhibition, low recurrence, and high survival rate [117]. The IS also exhibited hemostatic function and good pH sensitivity.

Another material base, silica nanoparticles (SiO_2_ NPs), is evolving in hydrogel research due to their biocompatibility, large surface area, and the possible control of their morphology. De Melo Santana et al. reported a Pluronic F-127/hyaluronic acid hydrogel containing nitric oxide (NO) donor S-nitrosoglutathione (GSNO) and silica nanoparticles loaded with cisplatin (SiO2@CisPt NPs) as a drug-delivery approach for sustained and localized drug release against tumor cells [118]. A synergistic toxicity of GSNO and SiO2@CisPt on breast cancer cells represented by pronounced cell death was observed.

Bombyx mori silk fibroin (BMSF) and Antheraea assamensis silk fibroin (AASF) hydrogels were also used for localized drug delivery in TNBC therapy post-lumpectomy [119]. This system, loaded with dexamethasone, exhibited sustained release of doxorubicin to target cancer cells and support differentiation of ADSCs along with vascularization. Additionally, this system was tested in a 3D in vitro lumpectomy model, which was developed using the MDA-MB-231 cancer cell line, and exhibited potential to reduce tumor recurrence and drug toxicity and support adipose tissue regeneration for breast restoration.

With the concept of introducing a dual-effect of chemotherapy and tissue regeneration, Balahura et al. created cellulose nanofiber (CNF)-based hydrogels incorporating 5-fluorouracil (5-FU) and alginate/pectin (A.CNF or P.CNF) [120]. This platform showed improved biocompatibility and cellular properties when pectin was dispersed within CNFs, effectively suppressing breast cancer cell growth and inducing pyroptosis. The scaffolds supported the growth of human adipose-derived stem cells, suggesting a role in soft tissue reconstruction following mastectomy, a potential awaiting to be tested in animal models before translational applications.

Mifepristone combined with paclitaxel could be an effective strategy for inhibiting breast cancer metastasis. However, a short half-life in blood circulation and lack of tumor targeting limit their effectiveness and cause adverse reactions. In a study by Zhao et al., paclitaxel (PTX)-conjugated and mifepristone (MIF)-loaded succinic anhydride hydrogel (PM-nano) was prepared [121]. They found that this hydrogel provides effective drug delivery and biocompatibility in mice and in vitro. The combination of PTX and MIF in PM-nano suppressed the expression of metastatic cancer biomarkers (ROR1, MMP2, and MMP9). These recent drug delivery hydrogel models, representing a portion of existing hydrogel types that can deliver drugs, collectively showed the diversity, flexibility, capacity, and compatibility of the engineered hydrogels in incorporating chemotherapeutic drugs for treatment testing.

### 4.2. Hydrogel Delivery of Immunotherapy and Chemoimmunotherapy

Cancer immunotherapy involves the use of a medicine to improve a patient’s own immune functions to eliminate cancer cells. It is used to treat locally advanced or metastatic breast cancer. The current immunotherapeutics commonly fall into five categories: monoclonal antibodies (targeted cancer drugs), checkpoint inhibitors, CAR T-cell, vaccine, and cytokine. There is a limited report on clinical outcomes of immunotherapy on breast cancer patients and so is hydrogel-based immunotherapeutics delivery testing in in vivo models. Yet, there is encouraging research ongoing to fill up the gap. As mentioned above in hydrogel delivery of chemotherapeutics, the MGC hydrogel generated by Seo and colleagues was shown to act as an antigen source for generating a host antitumor immune response by mediating immunogenic cell death (ICD), which presumably induced the recruitment and activation of antigen-presenting cells and activating cytotoxic T lymphocytes [115].

In an in vitro study conducted by Sultan et al., two hydrogels were synthesized: cisplatin-loaded chitosan nanoparticles (CCNP) and cisplatin-loaded chitosan nanoparticles surface-linked to rituximab (mAbCCNP) [122]. Here, rituximab, which is a monoclonal antibody acts to treat cancers via directly binding to CD20 antigen on B cells [123], was used with the intent of enabling the gel to bind to MCF-7 breast cancer cells with higher affinity, not as a cytotoxic therapy. After administration of the gels, however, the authors found that although the rituximab-linked gel (mAbCCNP) could bind to human breast cancer cells, it did not exhibit expected cytotoxic effects due to insufficient targeting, limited specificity, inadequate drug release, and possible cellular resistances.

Recently, Mantooth and Zaharoff generated an XCSgel by cross-linking chitosan that was used to contain interleukin-12 (IL-12) for injection into TNBC tumors in mice [124]. They found that formulated XCSgel-IL-12 delivery eliminated the tumors and stimulated immune memory, suggesting a neoadjuvant treatment potential of the strategy prior to breast-conserving surgery.

Integrating immunotherapeutics into chemotherapeutic regimens can potentially better combat advanced breast cancers compared to using chemo drugs alone. For instance, in a clinical trial, breast cancer patients with relatively high levels of programmed death ligand-1 (PD-L1) protein expression had a close to 43% improvement in overall survival after treatment with both pembrolizumab (Keytruda), an immunotherapeutic monoclonal antibody targeting and blocking the programmed cell death protein 1 (PD-1) receptor on lymphocytes for the protected immune system to attack cancer cells, and chemotherapy compared to those only received chemotherapy [125]. In another randomized clinical study, TNBC patients who received pembrolizumab, in addition to chemotherapy, preoperatively showed better outcomes than patients who only received the chemotherapy with a placebo [126]. However, the muti-chemo regimen is rather toxic to patients. The body conditions of certain patients may not allow for such strong therapeutic combinations, and the overall life quality of the patients could be compromised as a tradeoff of combination therapy.

**Figure 2:**
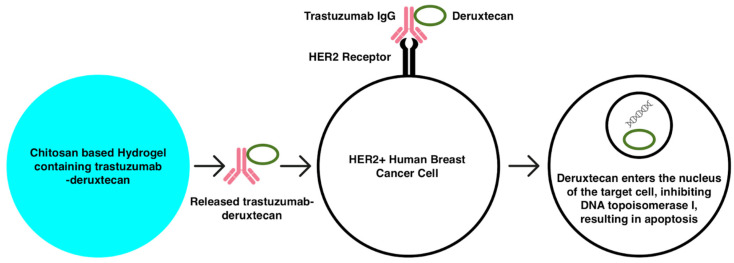
The theoretical mechanism by which trastuzumab–deruxtecan enters and induces cell death in HER2+ human breast cancer cells is shown.

Trastuzumab–deruxtecan (Enhertu) is an antibody–drug conjugate (mixed therapy) [127] for HER2+ breast cancer patients. Trastuzumab, the immunotherapy portion of this mixed therapy, binds to the extracellular domain of HER2 with relatively high affinity [128]. This binding inhibits the growth and the proliferation of HER2+ cells by the delivery of the chemotherapy portion of the conjugate, deruxtecan—an exatecan derivative that kills cancer cells by inhibiting DNA topoisomerase I [129] (Figure 2). The DESTINY-PanTumor02 Phase II clinical trial of the trastuzumab and deruxtecan drug conjugate illustrated significant clinical survival outcomes such as overall survival benefits in patients with HER2+ solid tumors [130]. In a study conducted by Gréa et al., spatiotemporal release of monoclonal antibodies including trastuzumab and rituximab was shown to be successful via the use of a chitosan-based hydrogel [131].

Cai et al. applied an injectable Pluronic F-127 hydrogel, made of polyethylene oxide and polypropylene oxide [132], infused with chemoimmunotherapeutics triptolide and IFN-γ to treat TNBC in mice [133]. The injected therapeutic hydrogel was shown to reverse IFN-γ-inducible PD-L1 expression and activate antitumor immunity in vivo. Moreover, the treatment led to a significant increase in the amount of CD8+ helper T cells in the spleen when compared to either drug used in isolation. This suggests an enhanced activation of antitumor immunity as a result of the local hydrogel delivery of the chemoimmunotherapy.

### 4.3. Hydrogel Delivery of Other Cancer Therapies

Although the mainstay treatment for breast cancer involves surgery, chemotherapy, and immunotherapy, other therapies, especially hormone therapy, are widely applied in breast cancer treatment [134]. In a study on tamoxifen performed by Mondal et al., a temperature-sensitive hydrogel (Tam-Gel) was developed. The Tam-Gel exhibited a transformation from liquid to gelatin at room temperature and another transformation when exposed to body heat, releasing tamoxifen in a well-controlled way. This room-to-body heat temperature transformation of the Tam-Gel hydrogel makes it a good candidate for controlled delivery of tamoxifen, as well as other hormonal and non-hormonal therapeutics [135].

Photothermal therapy (PTT) is an antineoplastic method that involves using electromagnetic radiation to treat cancer. This process delivers energy directly into the tumor mass that is skin penetrating near-infrared irradiation (NIR). This laser delivered to the targeted tumor cells can induce localized photochemical, photomechanical, and photothermal reactions that can kill the cells [136]. With this therapy, Yang et al. developed an injectable hybrid hydrogel platform (IR820/Mgel) that integrates indocyanine green (IR820) and MPs for photothermal therapy of breast cancer [137]. IR820/Mgel exhibited rapid heating above 50.0 °C under near-infrared (NIR) irradiation, effectively killing 4T1 breast cancer cells in vitro and preventing post-surgical tumor recurrence in vivo. This platform was minimally invasive and capable of filling irregularly shaped defects post-surgery, with the MPs enhancing gel strength for sustained in situ function [137].

Qi et al. investigated photothermal photodynamic therapy using bovine serum albumin-modified molybdenum disulfide nanoflakes (BSA-MoS2 NFs), which were loaded in an injectable polysaccharide hydrogel [138]. The nanocomposite hydrogel had significant photothermal conversion properties and generated reactive oxygen species under 808 nm NIR laser irradiation. In vivo anticancer studies indicated that the hydrogel can be directly injected into tumors and can remain there to achieve synergistic antitumor photothermal-photodynamic therapeutic effects.

Combining photothermal therapy with immunotherapy, Shen et al. developed an injectable copper (Cu)-induced hydrogel combined with a nitric oxide (NO) donor and PD-L1 antibody [139]. The hydrogel had persistent photothermal effects upon NIR laser irradiation, inducing immunogenic cell death (ICD) and modulating the TME to promote immune cell infiltration and reduce immunosuppression. The authors’ in vitro and in vivo studies demonstrated the hydrogel’s ability to enhance therapeutic efficacy and decrease tumor recurrence.

With a focus on integrating photothermal therapy along with tissue regeneration, Luo et al. utilized a 3D-printed scaffold composed of dopamine-modified alginate and polydopamine (PDA) for breast cancer therapy and tissue repair [140]. This scaffold exhibited NIR-induced photothermal effects and demonstrated flexibility and modulus like those of native breast tissues. It promoted the adhesion and proliferation of normal breast epithelial cells and supported tissue repair post-surgery. The scaffold’s performance could be tracked using magnetic resonance and photoacoustic dual-modality imaging, underscoring its potential for clinical treatment of breast cancer [140].

Li et al. created a self-healing hydrogel incorporating graphene nanoparticles and chondroitin sulfate multialdehyde (CSMA), along with branched polyethyleneimine (BPEI) and BPEI-conjugated graphene (BPEI-GO), for preventing postoperative recurrence of breast cancer [141]. This hydrogel combined regeneration, photothermal therapy, and chemotherapy into one system and demonstrated excellent self-healing properties and mechanical strength, which facilitated sustained drug delivery and NIR-triggered photothermal effects. In their mouse models, a combinational therapy using Doxorubicin (DOX), and photothermal therapy showed significantly reduced tumor recurrence compared to controls. This implicates the potential of applying the CSMA/BPEI/BPEI-GO hydrogels for effective breast cancer therapy and demonstrates the capability of hydrogels in incorporating multifaceted treatment methods.

Curcumin is a polyphenol extracted from the rhizomes of the turmeric plant, Curcuma longa, which has anti-inflammatory and anticancer properties via its action on the regulation of various immune modulators, including cytokines, cyclooxygenase-2, and reactive oxygen species. Clinical trials of curcumin are either completed or ongoing for various types of cancer. Shpaisman et al. studied a curcumin-derived hydrogel cross-linked with carbonate linkages for targeted anticancer drug delivery [142]. The hydrogel incorporated curcumin into its polymer backbone, protecting it from oxidation and degradation. Controlled release studies showed selective cytotoxicity against the MDA-MB-231 breast cancer cells but not quiescent human dermal fibroblasts. This model may be applicable as a filler for post-surgical breast tissues, demonstrating its potential in both therapeutic and reconstructive applications.

Hydrogel-based anticancer drug delivery approaches, though have started emerging in clinical trials, are still in an infant phase of development. The current clinically tested hydrogel delivery systems are mostly applied locally at or around the lesion sites. The benefit of systemic delivery of drugs with hydrogels is not clear yet. Though controlled drug release is an advantage of hydrogel-based drug delivery models, the long-term benefits of the delivering methods and the side effects associated with the hydrogel materials or their degradation products on human patients remain to be revealed in the coming years of studies. Additionally, only limited drug types have been tested in hydrogel delivery in animals or clinical trials. Whether hydrogel delivery is suitable for any given drug is waiting to be tested. Hydrogel material selections, delivery methods, drug doses, and drug-loading/releasing capacities of the hydrogels need to be studied in more detail, optimized, and standardized over time.

### 4.4. Regulatory Status of Hydrogels for Breast Cancer Therapy

With regard to cancer therapy, injectable hydrogels are the mainstay for use as drug delivery matrices [54,55]. Two hydrogel types with one using polyethylene glycol have been FDA- and EMA-approved for prostate cancer treatment. The other gel is made with hydroxyethyl methacrylate and hydroxypropyl methacrylate and cross-linked with trimethylolpropane trimethacrylate to release histrelin acetate following subcutaneous injection for palliative treatment of prostate cancer [54,55]. Outside of FDA regulation, various hydrogels are in clinical trial testing for cancer applications ranging from cervical, colorectal, head and neck, and bladder cancers. Most of these are injectable systems loaded with antineoplastics such as irinotecan and cisplatin [55].

For breast cancer application, one hydrogel tested in a clinical trial with an identifier NCT04481802 is a high molecular polysaccharide hydrogel called RadiaAce that is obtained from Aloe Vera gel [55]. This gel functions as a topical agent in preventing radiation dermatitis. Several other studies involving pain management and radiation-induced skin toxicities were found with a search through ClinicalTrials.gov. However, there are currently no active clinical trials of hydrogels being directly applied for treating breast cancer, such as with antineoplastics. There are homologous systems that are FDA-approved and in clinical trial testing. One hydrogel made from Pluronic F-127, PEG-400, and hydroxypropyl methylcellulose has been FDA-approved for the treatment of low-grade upper tract urothelial cancer in 2020 [54,55,143]. This has similarities to the system by Cai et al. for triple-negative breast cancer using co-delivery of IFN-γ and triptolide with a Pluronic F127 thermogel system to activate antitumor immunity [133]. The Cai’s system also shows similarities with a clinical trial-tested hydrogel called TumoCure with an identifier NCT05200650 which is a bulk polymer-based gel loaded with cisplatin for local injection to head and neck tumors [55]. Hydrogel delivery of breast cancer therapy may follow similar directions for regulatory approval in the clinical setting.

## 5. Conclusions

Hydrogel implants represent a burgeoning frontier in breast cancer therapy involving drug delivery and/or post-mastectomy reconstruction. The advancements in natural and synthetic materials and in bioengineering technologies have triggered diverse clinical needs for breast cancer treatment. Natural hydrogels, such as collagen or gelatin derivatives like GelMA, are able to resemble the native breast tissue microenvironment. The biomimetic properties of biomaterials support adipogenesis and vascularization while offering tunable mechanical properties through innovative cross-linking techniques [7,8,11,15,16]. Synthetic hydrogels, including polymers like PCL and PHEMA, provide more enhanced mechanical stability and durability due to their synthetic nature [6,25,26,27,28]. With applications to breast tissue restoration techniques, these hydrogel platforms play pivotal roles in enhancing surgical outcomes post-lumpectomy or mastectomy. They facilitate adipogenesis and vascularization, essential for tissue regeneration and aesthetic outcomes [47,48,50,51]. Natural scaffolds derived from ECM components and decellularized tissues provide a supportive environment for cell populations crucial in tissue engineering, promoting integration and functional restoration [46]. Innovative approaches combining collagen microfiber bio-inks with stem cells and endothelial cells demonstrate promising results in generating vascularized adipose tissues resembling native breast tissue structures [48]. Moving forward, the clinical translation of these advancements hinges on overcoming challenges such as degradation rates, biocompatibility concerns, and the need for scalable manufacturing processes [11,38]. The lack of direct hydrogel applications for breast tissue regeneration outside of silicone implants prompts the need for more development of these hydrogels [54,55]. Further research into refining hydrogel properties and optimizing scaffold designs will be crucial for further applications in personalized breast cancer therapies and reconstruction strategies.

Aside from tissue reconstruction, hydrogels, with proper formulation, are suitable tissue surrogates for studying patient-specific cancer characteristics, particularly in the realm of assessing treatment efficacy. 3D hydrogel models for breast cancer can replicate the tumor microenvironment (TME) using bioactive ECM or ECM-mimicking components like chitosan, alginate, and collagen [56,57]. Collagens, particularly type I, are abundant in breast tissues and essential for ECM structure and function, making them key in creating realistic 3D culture platforms [12,58,59,60]. Recombinant spider silk and enzymatically cross-linked silk fibroin hydrogels have shown promise for studying cancer progression and drug responses [68,70]. Incorporating immune cells and PDO into these models enhances their biological relevance and utility in drug screening, revealing nuanced responses to treatments and maintaining cellular characteristics [71,73,74,144]. The advances in 3D modeling, such as the use of ELR hydrogels and PDS, support the study of chemo and hormone therapy resistance, which highlights the importance of TME properties in drug efficacy tests [69,70,74]. Since native breast ECM protein compositions and structures, which are biological ligands and mechanical supports, respectively, are different from collagen, BME, and non-biological polymers and instructive to native cell receptor expression and phenotypes, incorporation of native ECM components into breast cancer biological studies is necessary. Future refining and standardizing the various hydrogel models for breast cancer modeling will benefit by incorporating more patient-specific features and enhancing their precision for personalized medicine and preclinical drug evaluation [99].

Hydrogels have the potential to be used as versatile platforms for the delivery of diverse antineoplastic drugs and even combined with tissue regeneration properties. Chitosan-based hydrogels showed promise in sustained drug release and enhanced hemostasis [115,116]. Silk fibroin hydrogels and 3D-printed PLGA scaffolds also demonstrated effective localized drug delivery and potential for post-resection breast reconstruction [117,119]. Silica nanoparticle-enhanced hydrogels loaded with cisplatin and nitric oxide donors exhibited synergistic toxicity against breast cancer cells [118]. Additionally, hydrogel systems, such as those incorporating paclitaxel and mifepristone, or combining chemoimmunotherapy agents like pembrolizumab and IFN-γ, demonstrated potential in reducing tumor metastasis and enhancing immune responses [121,133]. Other promising approaches include photothermal and photodynamic therapies using hydrogels with indocyanine green and molybdenum disulfide nanoflakes with significant antitumor effects [116,138]. FDA approval and clinical trial testing for these hydrogels specifically in breast cancer therapy are lacking, prompting further research [54,55]. Future directions should focus on optimizing these multifunctional hydrogels for the clinical treatment of breast cancer, improving their biocompatibility, and enhancing their ability to deliver combined therapies with precision, less toxicity, and more efficacy.

## Figures and Tables

**Table 1:** Survey of hydrogels recently studied for breast tissue reconstruction. Comparison of recent studies on natural and synthetic hydrogels used for breast tissue engineering with respect to material sources, composition, and features of the hydrogel studied.

Source	Material	Modification	Combination	Study Type	Advantages	Disadvantages	Ref.
Natural	Collagen/Gelatin	GelMA	GelMA/CS	In vivo	Versatility Adipogenesis Vascularization Porosity	Degradation Biocompatibility Mechanical stability under physiological stress High cost	[17]
Natural	Collagen/Gelatin	GelMA	GelMA/CarMA	In vitro	Cell viability Proliferation rate Structural stability Mechanical properties like breast	Poor differential potential compared to GelMA Uncertain stability	[19]
Natural	Collagen/Gelatin	f-GelMA	-	In vitro	High bio-compatibility Adipogenic differentiation Enhanced vascularization Minimal cell damage Higher cell survival rates	Integration complexity Need for further validation Technical challenges Uncertain long-term stability and functionality in vivo Scalability	[21]
Natural	Collagen/Gelatin	GelNB GelSH	-	In vitro	Cell-interactivity Reduced phase separation risk Homogeneous network formation Better physicochemical characteristics Enhanced adipogenic differentiation potential	Scalability Uncertain long-term stability and functionality in vivo Vascularization complexity	[22]
Natural	Collagen/Gelatin	Gelatin	Gel/alginate	In vitro	Mimicked tissue architecture Pre-vascular channels Cytocompatible method Mechanical mimicry Successful differentiation In vitro blood flow	Vascularization complexity Scalability Long-term stability	[23]
Synthetic	PHEMA	-	Maleic acid	In vitro/In vivo	High tensile strength High fracture strain Compliance similar to silicone breast prosthesis Self-recovery ability Fatigue resistance Biocompatibility in vivo	Fabrication complexity Limited translational validation Long-term durability and stability in clinical use	[36]
Synthetic	Polyvinyl/Alcohol	-	PVA/COL/PPG	In vitro	Improved mechanical properties Enhanced thermal stability Enhanced cell adhesion sites Three-dimensional cell growth support	Fabrication complexity Variability in scaffold performance Long-term stability in clinical use	[37]

## Data Availability

Not applicable.
